# Hoyt’s Practical Therapeutics

**Published:** 1915-02

**Authors:** 


### Book Reviews.

Hoyt's Practical Therapeutics. Published by C. V. Mosby Co., of St. Louis. Price, $5.00.

We desire to call the busy practitioner's attention to the new and revised edition of Hoyt's Practical Therapeutics. To the close student of medicine, as well, it will be found invaluable. At a glance the reader can get the drug, its physiological action—both special and general, its therapeutic indications and contra-indications; its toxicology and treatment. It contains a description and the use of all new and non-official drugs that have been passed upon by the Pharmacy Council of the A. M. A. It contains a chapter on Proprietary and Patent Medicines, and points out the value and importance of sticking to the official drugs whenever possible; it tells the dispensing physician what new drug can be satisfactorily used and relied upon, and as to its chemical composition. It contains both a therapeutic and medical index.

